# Supplementary material for: Learning curve and surgical outcome of robotic assisted colorectal surgery with ERAS program
Source: Sci Rep. 2022 Nov 29;12:20566. doi: 10.1038/s41598-022-24665-w (PMC9709162; doi:10.1038/s41598-022-24665-w)
Supplement: Supplementary file 2 — Supplementary Information 2. [file 41598_2022_24665_MOESM2_ESM.zip › RAL Raw data-Ñ[▒K/083/HS_ñw╝╨░O▒Kñσ.pdf]

正式摘要(電子簽章：000080217257967)		
W52 / W52 ☐英文標題(注意:無英文姓名-請補病人基本檔!)	☐【讀取】憑證	

臺中榮民總醫院
出院病歷摘要

轉入號： 轉出號：

[redacted]

(8)流水編號	
(9)入院日期	110/05/13 (2021)
(10)轉科日期	
(11)出院日期	110/05/18 (2021) 住院天數計 5 日

診斷

- 入院 :rectal cancer, L/3
- 出院 :1. Rectal cancer, L/3, s/p Robot Assisted Total mesorectal excision + loop ileostomy on 2021/05/14
  2. fever with chillness, suspected minor leak, s/p Curam

主訴

- rectal cancer admitted for Robot Assisted Low Anterior Resection.

病史

- The 63-year-old male had underlying disease of DM, HT, hyperlipidemia, PPU 7-8 years ago.
  He suffered from frequent flatus and blood tinged stool for 3-4 months. He went to 苗栗大千hospital, where coloscopy was performed, s/p biopsy and showed rectum cancer. CT was arranged there but he didn't receive it and went to our OPD for help. Digital examintaion showed palpable tumor, anterior wall at L/3. Therefore, under the impression of rectal cancer, he was admitted for Robot Assisted Low Anterior Resection.

體檢發現

- Anal inspection:
  Recto digital examination:PLAPABLE TUMOR, ANTERIOR WALL
  - General appearance :
    - easy looking , conscious clear , without cardiopulmonary distress
    - Vital signs自動帶入date 體溫(BT) 脈搏(P) 呼吸(R) 血壓(BP) 身高(BH) 體重(BW) BMI
      20210513 169.5Cm 71.8Kg 25.0
    - Vital signs: B.P. 136/94 mmHg , P.R. 108 /min , R.R. 18 /min , B.T. 37 ℃
  - HEENT :
    - Eyes: no anemic conjunctiva, no icteric sclera, normal EOM
    - ENT: no otorrhea, no rhinorrhea, no erythematous mucosa, no injected tonsil, no deformity
  - Neck: supple, freely movable, trachea midline,no deformity, no engorged jugular vein , no palpable thyroid or lymph node enlargement
  - Chest:
    - Inspection: Grossly normal, symmetric and fully expansion, no accessory muscle used, no operation scar, no spider angiomata, no gynecomastia
    - Palpation: no palpable mass or nodule, no subcutaneous emphysema, no tenderness
    - Ausculation: clear breathing sound
  - Heart:
    - Percussion: normal heart size
    - Ausculation: regular heart beat, normal S1, S2, no gallop, no murmur
  - Abdomen:
    - Inspection: in
      - flat contour, symetrically, no scar, no stria, no dilated veins, no rashes
    - Ausculation: normoactive bowel sound, no abdominal bruits
    - palpation: soft, no muscle guarding or rigidity, no local tenderness, no rebounding pain, no palpable mass, no palpable pulsation
    - Percussion: no shifting dulllness, no hepatomegaly, no splenomagaly
  -

Peripheral pulsation :	CA	BA	RA	FA	PA	PTA	DPA
Right side :	++	++	++	++	++	++	++/+++
Left side :	++	++	++	++	++	++	++/+++

  - Genito-anus: no tenderness or palpable mass lesion or rectal shelf, normal external genitalia, normal anal tone, brown stool

CRS CONDITION

-
  - Lung

手術日期及方法

- 手術日期及方法20210514 Robot Assisted Low Anterior Resection + LOOP ILEOSTOMY

住院治療經過

- After admission, pre op survey was OK and operation was done smoothly. He tolerated the procedure well and could stand oral feeding gradually. Chillness and fever was noted on 05/15 which subsided under Abx. Under relatively stable conditions, he will be discharged.

合併症

一般檢查

- 放射線報告 :**檢驗項目 :CT ABDOMEN-SURVEY 報告日期 :1100514**
  主 訴：

- Result:Y
  Finding:
  Imaging Report Form for Colorectal Carcinoma (AJCC 8th)
  1. Imaging Date: 20210513
  Imaging Modality, Imaging by
  CT scan
  Imaging Protocol: CT scan of abdomen with IV contrast enhancement:
  2. Tumor location / Size
  Location:
  Rectum
  Size:
  Measurable: 4 cm (largest diameter)
  3. Tumor invasion
  Tumor invades non-peritonealized pericolonic or perirectal tissues
  4. Regional nodal metastasis
  No
  5. Distant metastasis (In this study)
  No or Equivocal
  6. Other findings: > History of rectumr cancer for pre-operation survey
  > Asymmetric wall thickening of rectum at least 4cm in length with stranding of perirectal fat plane, C/W rectum cancer (Axi:77-85, Cor:34-40)
  > Mild fatty liver
  > A renal cyst over right kidney
  > Bilateral hydrocele
  > Presence of hiatal hernia is noted.
  > The pancreas, spleen, gallbladder, left kidney are without specific abnormality
  > No significant lymphadenopathy in the abdomen or retroperitoneum is identified
  > No osteolytic or osteoblastic bone lesion is identified in visible bony structure.
  Impression:
  1. R/I rectum cancer. Please correlate with colonoscopy.
  2. For other details, please see the above descriptions.

特殊檢查

病理報告

其它

出院時情況

- 3.改門診治療

出院指示

藥名	劑量	劑量單位	用法	頻次	數量	數量單位	現況	天數	單位
(口)Takepron OD tab 30mg	1	TAB	PO	QDAC	8	TAB	TH	8	天
Mag. Oxide tab	2	TAB	PO	TID	48	TAB	TH	8	天
Ultracet tab	1	TAB	PO	Q6H	32	TAB	TH	8	天
Curam Film-coated tab 1000mg	1	TAB	PO	Q12H	14	TAB	TH	7	天
(凵)Rosis tab 40mg	1	TAB	PO	QD	8	TAB	TH	8	天

出院計劃

主治醫師 2062F 蔣鋒帆 / 住院醫師 2309E 古朋育

正式摘要(電子簽章：**000000217257967)**

W52 / W52

doctorRank:B
